# Supplementary material for: Supermarket Circulars Promoting the Sales of ‘Healthy’ Foods: Analysis Based on Degree of Processing
Source: Nutrients. 2020 Sep 21;12(9):2877. doi: 10.3390/nu12092877 (PMC7551930; doi:10.3390/nu12092877)
Supplement: Supplementary file 1 [file nutrients-12-02877-s001.pdf]

Table S1. NOVA food groups: definition according to the extent and purpose of food processing, with examples

NOVA GROUP	DEFINITION	EXAMPLES
**1) Unprocessed or minimally processed foods**	**Unprocessed:** edible parts of plants (fruits, seeds, leaves, stems, roots, tubers) or of animals (muscle, offals, eggs, milk), and also fungi, algae and water, after separation from nature.<br><br>**Minimally processed:** unprocessed foods altered by industrial processes such as removal of inedible or unwanted parts, drying, crushing, grinding, fractioning, roasting, boiling, pasteurisation, refrigeration, freezing, placing in containers, vacuum packaging, non-alcoholic fermentation, and other methods that do not add salt, sugar, oils or fats or other food substances to the original food. The main aim of these processes is to extend the life of unprocessed foods, enabling their storage for longer use, and, often, to make their preparation easier or more diverse. Infrequently, minimally processed foods contain additives that prolong product duration, protect original properties or prevent proliferation of microorganisms.	Fresh, squeezed, chilled, frozen, or dried fruits and leafy and root vegetables; grains such as brown, parboiled or white rice, corn cob or kernel, wheat berry or grain; legumes such as beans, lentils, and chickpeas; starchy roots and tubers such as potatoes, sweet potatoes and cassava; fungi such as fresh or dried mushrooms; meat, poultry, fish and seafood, whole or in the form of steaks, fillets and other cuts, fresh or chilled or frozen; eggs; fresh or pasteurized milk; fresh or pasteurised fruit or vegetable juices (with no added sugar, sweeteners or flavours); grits, flakes or flour made from corn, wheat, oats, or cassava; tree and ground nuts and other oily seeds (with no added salt or sugar); herbs and spices used in culinary preparations, such as thyme, oregano, mint, pepper, cloves and cinnamon, whole or powdered, fresh or dried; fresh or pasteurized plain yoghurt; tea, coffee, and drinking water. Also includes foods made up from two or more items in this group, such as dried mixed fruits, granola made from cereals, nuts and dried fruits with no added sugar, honey or oil; pasta, couscous and polenta made with flours, flakes or grits and water; and foods with vitamins and minerals added generally to replace nutrients lost during processing, such as wheat or corn flour fortified with iron and folic acid.

**2) Processed culinary ingredients**	Substances obtained directly from group 1 foods or from nature by industrial processes such as pressing, centrifuging, refining, extracting or mining. Their use is in the preparation, seasoning and cooking of group 1 foods. These products may contain additives that prolong product duration, protect original properties or prevent proliferation of microorganisms.	Vegetable oils crushed from seeds, nuts or fruits (notably olives); butter and lard obtained from milk and pork; sugar and molasses obtained from cane or beet; honey extracted from combs and syrup from maple trees; starches extracted from corn and other plants, and salt mined or from seawater, vegetable oils with added anti-oxidants, and table salt with added drying agents. Includes products consisting of two group 2 items, such as salted butter, and group 2 items with added vitamins or minerals, such as iodised salt.
**3) Processed foods**	Products made by adding salt, oil, sugar or other group 2 ingredients to group 1 foods, using preservation methods such as canning and bottling, and, in the case of breads and cheeses, using non-alcoholic fermentation. Processes and ingredients here aim to increase the durability of group 1 foods and make them more enjoyable by modifying or enhancing their sensory qualities. These products may contain additives that prolong product duration, protect original properties or prevent proliferation of microorganisms.	Canned or bottled vegetables and legumes in brine; salted or sugared nuts and seeds; salted, dried, cured, or smoked meats and fish; canned fish (with or without added preservatives); fruits in syrup (with or without added anti-oxidants); freshly made unpackaged breads and cheeses.
**4) Ultra-processed foods**	Formulations of ingredients, mostly of exclusive industrial use, that result from a series of industrial processes (hence 'ultra-processed'), many requiring sophisticated equipment and technology. Processes enabling the	Carbonated soft drinks; sweet or savoury packaged snacks; chocolate, candies (confectionery); ice-cream; mass-produced packaged breads and buns; margarines and other spreads; cookies (biscuits), pastries, cakes, and cake mixes; breakfast 'cereals', 'cereal' and 'energy' bars; 'energy' drinks; milk

	manufacture of ultra-processed foods include the fractioning of whole foods into substances, chemical modifications of these substances, assembly of unmodified and modified food substances using industrial techniques such as extrusion, moulding and pre-frying, frequent application of additives whose function is to make the final product palatable or hyper-palatable ('cosmetic additives'), and sophisticated packaging, usually with synthetic materials. Ingredients often include sugar, oils and fats, and salt, generally in combination; substances that are sources of energy and nutrients but of no or rare culinary use such as high fructose corn syrup, hydrogenated or interesterified oils, and protein isolates; cosmetic additives such as flavours, flavour enhancers, colours, emulsifiers, sweeteners, thickeners, and anti-foaming, bulking, carbonating, foaming, gelling, and glazing agents; and additives that prolong product duration, protect original properties or prevent proliferation of microorganisms. Processes and ingredients used to manufacture ultra-processed foods are designed to create highly profitable products (low cost ingredients, long	drinks, 'fruit' yoghurts and 'fruit' drinks; 'cocoa' drinks; 'instant' sauces; infant formulas, follow-on milks, other baby products; 'health' and 'slimming' products such as meal replacement shakes and powders. Many ready to heat products including pre-prepared pies and pasta and pizza dishes; poultry and fish 'nuggets' and 'sticks', sausages, burgers, hot dogs, and other reconstituted meat products, and powdered and packaged 'instant' soups, noodles and desserts.

	shelf-life, emphatic branding), convenient (ready-to-consume) hyper-palatable snacked products liable to displace all other NOVA food groups, notably group 1 foods.	

Reference: Monteiro, C.A.; Cannon, G.; Levy, R.B.; Moubarac, J.-C.; Louzada, M.L.; Rauber, F.; Khandpur, N.; Cediel, G.; Neri, D.; Martinez-Steele, E.; et al. Ultra-processed foods: what they are and how to identify them. Public Health Nutr. 2019, 22, 936–941, doi:10.1017/S1368980018003762.

Table S2. Additional Claims (AC) in FoP labels of foods advertised in 'health and wellness' sections of supermarket circulars, classified by themes.

THEME	ADDITIONAL CLAIMS
FREE-FROM OR LOW IN LACTOSE	Free-from lactose
	Low in lactose
FREE-FROM GLUTEN OR WHEAT-FREE	Free-from gluten
	Wheat free
VITAMINS, MINERALS AND FATTY ACIDS	Vitamins
	Vitamin E
	Vitamin D
	Vitamin B2 + vitamin B12
	With vitamin C
	10 vitamins and minerals
	26 vitamins and minerals for everyday
	Calcium
	Calcium + magnesium + vitamin B complex, and vitamins A, C, and D
	Essential fatty acids
PLANT-BASED	Vegan
	100% veggie
	No animal ingredients
	Vegetable-based ingredients
PROTEIN	3.7g protein
	9g protein
	13g protein
	14g protein
	21g protein
	23g protein
	15g whey protein
	Whey isolate
	5g BCAAs
PROBIOTICS	Milk and probiotics
	Daily probiotic shot
	With active *Lactobacillus*
PRESENCE OF SWEETENERS	100% Stevia
	With natural Stevia
	Sweetened with Stevia
	Sweetened with sucralose
ORGANIC	Organic
NON-TRANSGENIC	Non-transgenic
	Soy-free
NATURAL	100% natural
	Natural salts
	Natural as it should be
	With natural ingredients
	Contains natural fibres and vitamins A and C

**LOW FAT CONTENT**	17% fat
	10% fat
	Only 1% fat
**WHOLE GRAIN AND FIBRE**	Whole grain
	Whole grain first ingredient
	With 14 grains
	Made with Granfibra®
	With cereals, oat, rice, wheat and corn
	With fibre
	Soluble fibre
**HIGHLIGHT ON THE PRESENCE OF INGREDIENTS**	With fruit flakes
	Pure fruit
	With fruit bits / pieces
	Sweetened only with sugar contained in fruits
	Only fruit, ready for the lunchbox
	100% grape juice
	Whole fruit juice / 100% fruit juice
	With brown sugar
	Oats and brown sugar
	24% oat
	With oats and chocolate chips
	With cocoa nibs
	With Saracen grains
	With rice, oats and flaxseed
	20% rice
	100% buffalo
	Coconut water with 100% Brazilian green coconut
	100% color and flavour from the tomatoes
	Made with almonds
	With nutrients from cane sugar plant
	With Himalayan pink salt
	Contains honey
	With cotton oil
**HIGHLIGHT ON THE ABSENCE OF INGREDIENTS**	No added preservatives
	No added water
	Zero / no sweeteners
	0% corn starch
	0% blah blah blah (referred not to have ingredients other than fruits)
**HEALTH AND NUTRITION**	Energy booster
	Pure energy
	Healthier
	Live healthier
	Have a healthier life
	Your healthier choice
	Nutrient rich
	More nutritious and tasty
	More full nutrition
	With nutritious grains
	Grains of goodness
	Balance and digestion

	Low fat absorption
	Contains beta-glucan
	Fruit with high antioxidant power
	Less processed
**CULTIVATION, PRODUCTION AND COOKING PRACTICES**	Baked
	Steamed
	Here we have family farming
	From the farm to your table
	Free range
	No added hormones or therapeutic antibiotics
	Fed with 100% vegetables
	Biological fermentation
	Made with ingredients you know
	Handmade
**BETTER QUALITY**	Gourmet
	Premium
	Superior quality
	Selected fruits
	With selected grains
**APPEAL TO SENSORY CHARACTERISTCS**	Experience the amazing flavor
	Crispier texture
	Great taste after every bite
	Aromatic and refined
	Refined and outstanding
	Dense and balanced
	Cereal letters *(cereals with letter format - visual appeal)*

Reference: Created by the authors (2020).

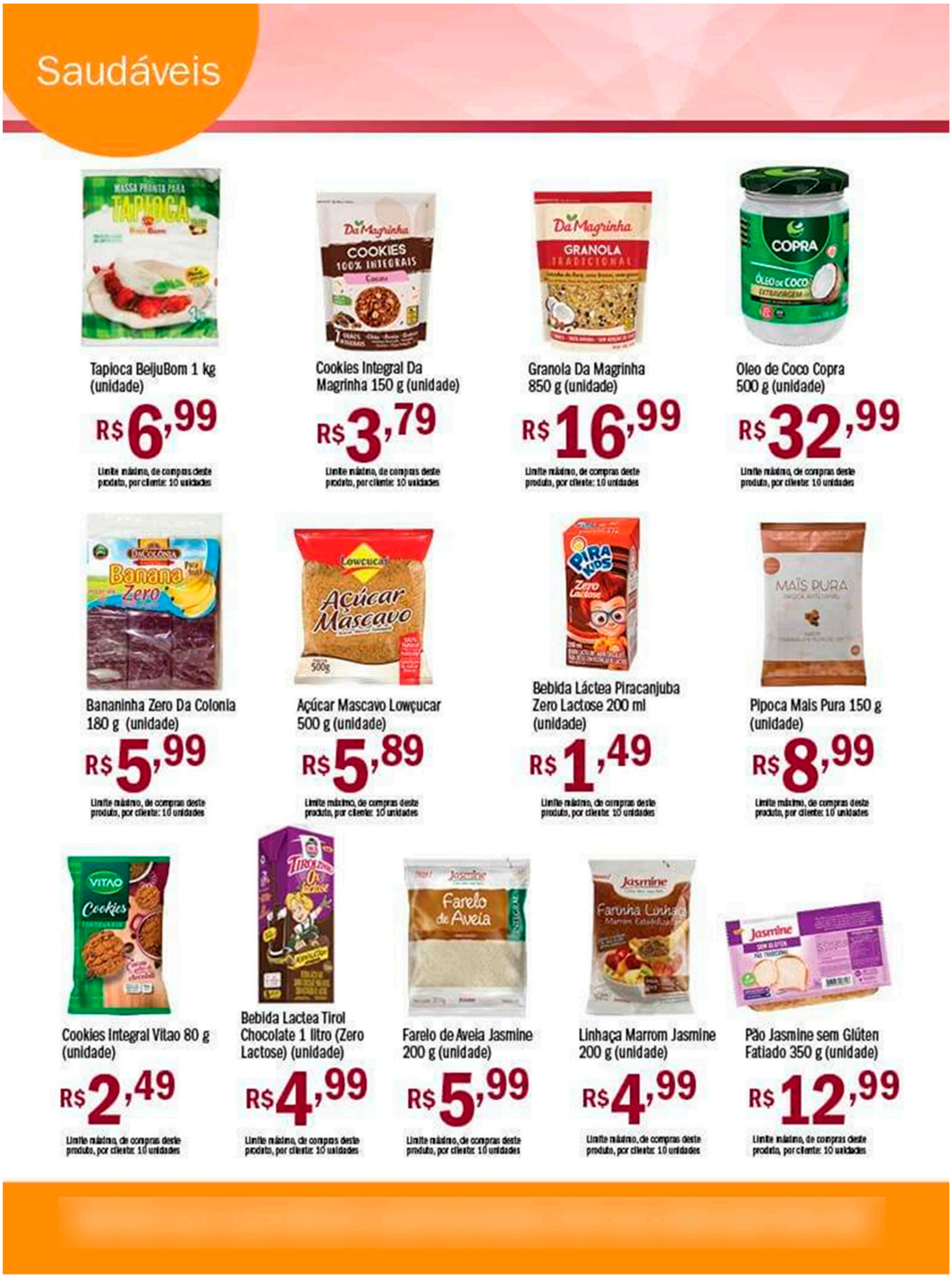

Figure S1. Examples of circulars' health and wellness sections (page 1)

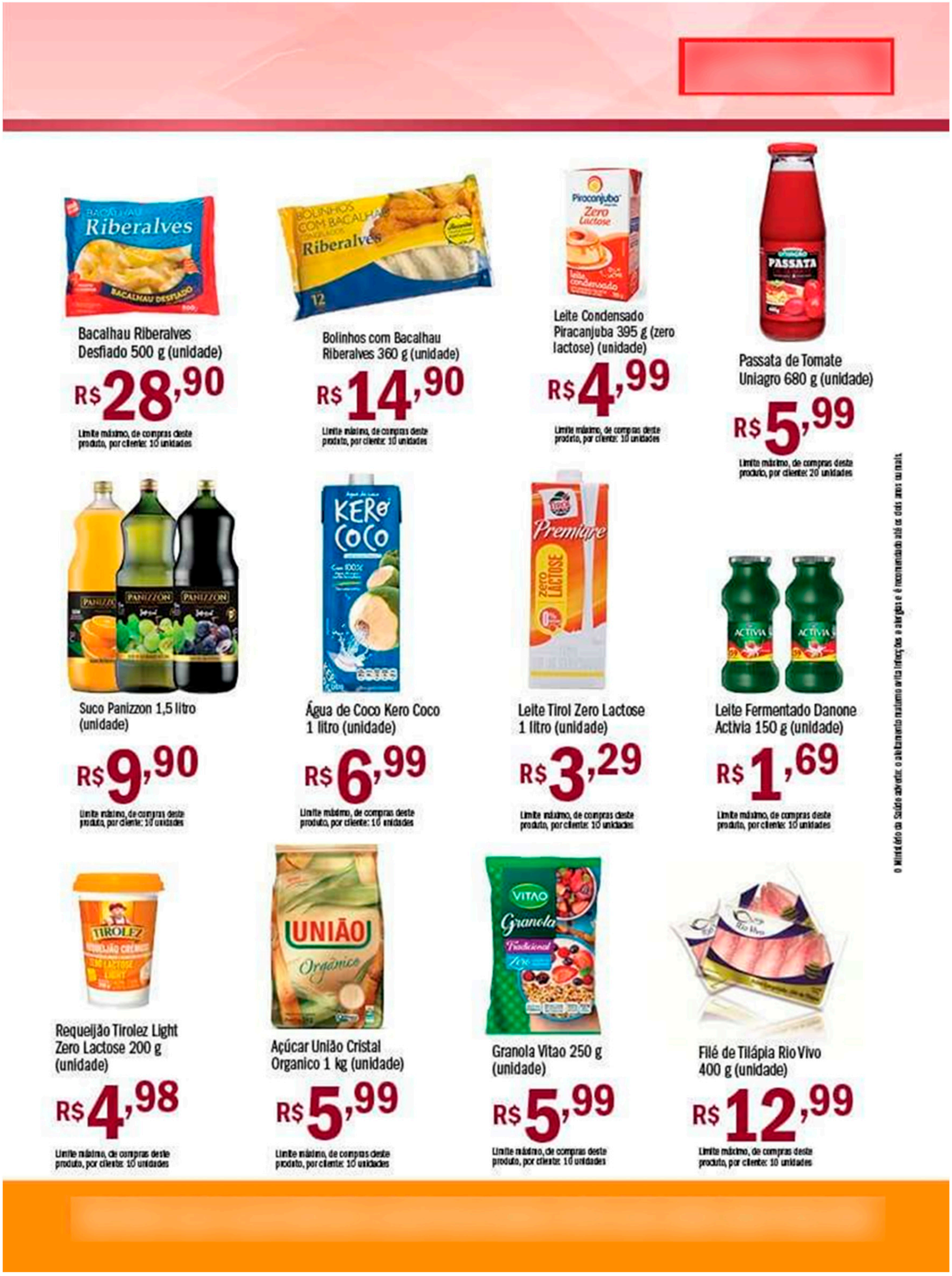

Figure S2. Examples of circulars' health and wellness sections (page 2)
